# Biopiracy: Abolish Corporate Hijacking of Indigenous Medicinal Entities

**DOI:** 10.1155/2021/8898842

**Published:** 2021-02-18

**Authors:** Yoonus Imran, Nalaka Wijekoon, Lakmal Gonawala, Yu-Chung Chiang, K. Ranil D. De Silva

**Affiliations:** ^1^Interdisciplinary Centre for Innovation in Biotechnology and Neuroscience, Faculty of Medical Sciences, University of Sri Jayewardenepura, Nugegoda, Sri Lanka; ^2^Department of Biological Sciences, National Sun Yat-Sen University, Kaohsiung, Taiwan; ^3^Institute for Combinatorial Advanced Research & Education (KDU-CARE), General Sir John Kotelawala Defence University, Rathmalana, Sri Lanka

## Abstract

Biopiracy as “a silent disease” is hardly detectable because it does not leave traces frequently. The corporate hijacking of food is the most important health hazard in this era; giant commercial enterprises are using intellectual property rights to patent indigenous medicinal plants, seeds, genetic resources, and traditional medicines. The new era of biotechnology relies on the genes of living organisms as raw materials. The “Gene Rush” has thus become similar to that of the old “Gold Rush.” Sri Lanka has been spotted in the top 34 biodiversity hotspots globally. Moreover, localized in the tropics, human generations in Sri Lanka have utilized the array of plant species for herbal treatments and treatment of diseases. Sri Lanka after its 30-year civil war is moving towards a solid growth and conservation of the environment which is a major component in a sustainable development where the conservation of biodiversity plays a significant role. In this paper, we present an overview of typical cases of global biopiracy, bioprospecting via introduction of cost-effective deoxyribonucleic acid (DNA) fingerprinting and international protocol with Private-Public-People Partnership concept as excellent forms of utilization of natural resources. We propose certain perspectives as scientists towards abolishing biopiracy and also to foster the fair utilization of natural resources; since the economy of most developing countries is agriculture based, the gross domestic product of the developing countries could be increased by enhanced bioprospecting via introduction of cost-effective DNA fingerprinting technologies and thus not being a pray of corporate hijacking.“Biopiracy is biological theft; illegal collection of indigenous plants by corporations who patent them for their own use” (Vandana Shiva).

## 1. Introduction

Nonetheless, the early European explorers sailed east and west in search of gold, silver, and rare spices, and medicinal plants were the treasure they found. In his book “The Customs of the Kingdoms of India,” Marco Polo (1254–1324) wrote “We shall tell you next of the great kingdom of Malabar [Southwestern India] there is a great abundance of pepper and ginger, besides cinnamon in plenty and coconuts.” Moreover, in the year 1293, Marco Polo sailed homeward bound from China, pausing at Seilan (Ceylon or Sri Lanka) along the way. He wrote “On leaving the island of Andaman and steering for 1,000 miles a little South of West, the traveller reaches the island of Seilan (Ceylon). The actual size is better circumstanced than any other island in the world” [1, 2].

The writings of Marco Polo were instrumental in the development of Spice Route [3] which was later utilized in colonial “Plant Hunting” that began in 1400 and peaked in the sixteenth and seventeenth centuries [4]. Following the Spice Routes, Christopher Columbus voyaged across the Atlantic Ocean with the blessings of the Spanish Court and sorted new route to the east through which wealth of knowledge on plant-based riches were obtained. Charlton noted that Columbus collected many valuable plants including Tobacco from Cuba in the year 1492 [5].

According to [3], the definition of biopiracy is “the unauthorized extraction of biological resources and/or associated traditional knowledge from developing countries, or the patenting of spurious inventions based on such knowledge or resources without compensation.” The hidden cases of biopiracy were started opposed, and the term biopiracy was coined in the 1990s by environmentalists and nongovernmental organizations [6]. Biopiracy as “a silent disease” is hardly detectable because it frequently does not leave any traces. Unfortunately, the electronic media favours to highlight environment pollution and deforestation, while biopiracy incidents are less reported. This silent pillaging is depriving countries that lack proper advancement in biotechnology primarily in Africa, Latin America, and Asia of the means to financially support developing and sustaining biotechnological projects. Biopiracy disrupts biodiversity conservation efforts [7–9].

The corporate hijacking of food is the most important health hazard in this era; using intellectual property rights, larger cooperation gets patent on indigenous medicinal plants, seeds, genetic resources, and traditional formulas by excluding local identity, as listed in Table 1. Taking advantage of these rights, “biopiracy” has happened by taking biological resources from one country to another country with the intention of building up global economies. As highlighted at the protest in the late 1990s by Dr. Vandana Shiva, the two examples from Indian context can be highlighted as basmati varieties of rice are transferred to build up the rice economy of United States and the export of neem seeds from Indian farms by giant corporates [15, 20].

According to [21], this undoubtedly indicates that European companies make enormous profits by using the biological properties of native and endemic plants from the developing countries but nothing goes back to them [21]. The share of the international trade of herbal products and alternative medicine market in a global economy has been increasing at a rapid rate of approximately 15% annually. Approximately 29,000 herbal substances used by more than 1000 companies have annual revenues exceeding US$ 60 billion, with the bulk of the herbal products, or at least the raw material sourced from biodiversity-rich countries in Asia, Africa, and South America [22, 23].

Thereby, biopiracy has long been a concern of developing nations with rich biodiversity. “Nagoya Protocol” has given a resolution of the controversy in bioprospecting, and solutions will lead to many researchers neglect by showing their burden of regulation to work [24].

## 2. Review Methodology

The review process was divided into three major steps: title, abstract, and document screening. The previous biopiracy cases and natural product authentication techniques are reviewed in this article. Literatures were searched in the world's acknowledged databases including PubMed, Medline, Scopus, Embase, and Springer. The search was based on the key words: Biopiracy, Plant Authentication, Plant Barcode, Plant Marker, Bioprospecting, and Microsatellite DNA marker, and 4011 publications were identified.

**Figure 1:**
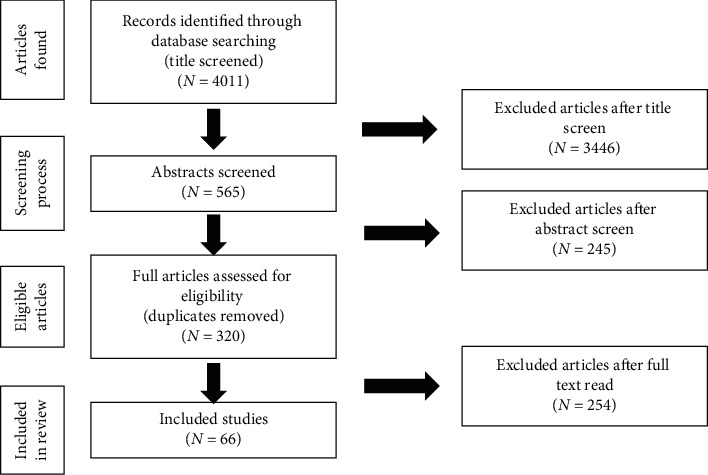
Method of article screening.

All the titles were screened, and 565 documents were downloaded for abstract screening. All 565 abstracts were screened, and 320 articles were retained which appeared to meet inclusion criteria: Biopiracy, Plant Authentication, Plant Barcode, Plant Marker, Bioprospecting, and Microsatellite DNA marker. Finally, the full text of all 320 retained documents was critically assessed using the same inclusion/exclusion criteria as the abstract screening, leaving 61 papers to be included in this review. Finally, reference lists of all 66 papers were inspected for additional relevant citations (see Figure 1).

## 3. Sri Lanka: A Biodiversity Hotspot

Sri Lanka has been spotted in the top 34 biodiversity hotspots globally, and it has the highest biodiversity per unit area of terrestrial among Asian countries. The wet zone rainforests are home to nearly all of the country's woody endemic plants [25]. Moreover, Sri Lanka has localized in the tropics and an range of plant species that have been utilized by human generations for herbal treatments and for treatment of diseases [26]. The 3 millennia old tested and proven efficacy of indigenous medicinal system in Sri Lanka is still in use by the locals. In the identified 1,500 species of medicinal plants in Sri Lanka, only a small percentage have been studied for their potential value as a source of drugs [27].

Biodiversity of Sri Lanka has a vast potential to be transformed as a source of funding. Most Multinational biotechnology companies rely on the genes of living organisms as its raw materials where they reiterate the old “Gold Rush” as new “Gene Rush” with the ultimate goal of future profits. Imminent danger to Sri Lankan biodiversity has been raised due to this quest for genetic resources among biopirates. Recent developments relating to biopiracy have been entered on a large alarming scale. Sri Lanka after its 30 years of civil war is looking towards sustainable development. Sustainable development must equip with the environment protection where conservation of biodiversity plays a significant role [28, 29].

Sri Lanka endows with a diversity of production of agriculture crops and is also rich in a range of spices including cinnamon, rubber, coconut, pepper, cardamom, cloves, nutmeg, and mace. The export of spices and allied products constitutes nearly 56% of the entire agricultural products. Especially Sri Lanka exports approximately 90% of true cinnamon to the world market and is the largest true cinnamon exporters in the world. Though Sri Lanka is owing to the worldwide demand for true cinnamon, pure cinnamon powder is often being adulterated with other inferior qualities. In a meeting for “Spicing up development assistance” in [30], United Nations Industrial Development Organization (UNIDO) agreed to help Sri Lankans to fulfill their vision of making Ceylon cinnamon as a one billion dollar industry [30–33].

## 4. Adulteration and Biopiracy: Economically Impact on Industries

A global survey shows that 27% of all successfully analysed commercial herbal products were tested with DNA-based analytical methods against their labelled, claimed, and expected composition, and all these adulterated products are distributed across all continents and regions [34].

## 5. The Approach to Mitigate Adulteration and Biopiracy

### 5.1. How DNA Markers Utilized for Plant Identification

The correct identification of plants is important in the nonscientific, commercial world as it is to ecologists and taxonomists. Genetic markers are broadly known as “DNA barcode forensics” and are being employed to ensure the identity and purity of commercial products in order to protect endangered species in illegal trading. A well-documented DNA barcode (rbcl and ITS2) helped to find that 59% of the products contained plant species not listed on the labels (many of them “fillers”) which has not only aroused attention in the scientific world but also made national news on New York Times. The news resulted in a backlash from the herbal supplement community concluded by recommending that the commercial herbal industry should routinely use DNA barcoding as a verification of the authenticity of constituents in all herbal products [35, 36].

Natural products are more competitive in the international market and increase consumer health, and now problems are encountered due to quality failures; i.e., Ceylon Cinnamon has a risk of losing the international market value due to the adulteration [31, 37]; true cinnamon stands out as the most expensive in the global market [38]. Adulterants from the genuine products in cases where there is a high physical resemblance between the entities, or in the instances of change in the physical form, could be discriminated using molecular markers [39].

Examining genetic diversity and lineage sorting of different genes in closely related species provides significant information for species delimitation and phylogenetic analysis. As genetic methods became more economical and accessible, the phylogenetic analysis made it possible to reveal hidden species diversity that was undetectable using morphological analysis [40].

DNA-based taxonomic identification has become an essential tool, and significant progress has been made on the improvement since the DNA barcoding concept was proposed [41]. DNA barcoding is a robust technique that uses a short sequence of DNA consisting of the invariable nucleotide sequence in subspecies but with sufficient divergence to discriminate between the species [42–44].

The developing countries are suffering to establish proper plant barcoding systems such as NGS, but they lack technology and economy. A high specific and cost-effective identification should be introduced in low- and middle-income countries where they could stop biopiracy in their territory to elevate the economy. Many low costs and simple barcoding methods have been proposed, but there is a conundrum for the cost-effective barcoding method for plant specimen identification [45, 46].

Given the above evidence, the barcoding methods established to date are not sensitive and efficient enough to provide noteworthy intraspecies deviations, which aid in identifying different species of plants such as cinnamon. Identification of microsatellites in plants could be a key to specify the plants and also clearly indicate the authenticity of species and the country. Microsatellite fingerprinting is based on high genetic variability and is one of the best molecular tools for elucidating the structure and genetic diversity of populations [47, 48].

Bioprospecting is a key method to overcome biopiracy, and the Nagoya Protocol has addressed and given solutions to major issues like the exploitation of traditional knowledge by innovations registered through the patent system. This is one of the main perceived injustices from biopiracy [49].

However, global governance to address biopiracy and uninterrupted sustenance of flora and fauna as exemplified by the recent Nagoya Protocol covered few fundamental issues faced by the locals and the lack of resources allocated for the management of biodiversity. The ethical, social, and political issues surrounding bioprospecting should be considered to protect the indigenous knowledge of traditional people from copyrighting corporate entities [50, 51]. For years, Private-Public Partnership (PPP) was used as an option to build agreements among organizations to stick on regulations for the transparent transactions, but greedy corporate entities and corrupted academics are still in the way of making profits by doing biopiracy. The aforementioned notion must be elaborated and acknowledged by all participants prior to any transaction of intellectual property transaction, and a reasonable compensation has been settled that befits the requirement of the locals [52].

Developing the involvement of private actors and the general public in a joint process could be archived by the concept of the Public-Private-People partnership (4P) which is an emerging approach in targeting attention to adding the general public to Public-Private partnerships and particularly addressing the problems of exclusion and lack of transparency. The 4P could be an ideal platform for crushing biopiracy with technological advancements [53].

Although individuals should not be able to make independent decisions about bioprospecting, nevertheless appropriate representatives of communities can have the authority to do so. 4Ps that employ corporate, indigenous, civil society organization partners, and individuals, as well as local researchers, should be held responsible in taking initiatives to formulate a system that paves the way for a mutual benefit. Via investments in 4P partnerships, all participants will hold equilateral intellectual property rights and responsibilities resulting in holding each other responsible for misconduct of a certain parties, and this also includes upcoming commercial endeavours. This concept if implemented as a best practise globally will promote the wellbeing of current and future populous [19].

### 5.2. Cost-Effective Genotyping

The actual choice of method must take into consideration on the marker availability, costs, expertise, equipment, and many other factors. Based on the papers published previously on discrimination among plant cultivars, locus-specific microsatellite analysis (SSR) was the most popular method (36%), followed by RAPD (27%), ISSR (13%), AFLP (11%), and other nuclear DNA-based methods (10%, including, for example, CAPS, DAMD, IRAP, REMAP, SNPs, SCAR, and SRAP) and organellar DNA-based methods (3%, mostly cpDNA) [54].

The genetic identification techniques summarized in Table 2 are currently being used for the plant identification with advantages and disadvantages in translational research. Further improvements and innovations of sequencing methodology can be expected and soon allow for cost-effective genotyping-by-sequencing of any nonmodel plant species. However, obtaining complete genomic sequence data for all individuals included in a study would be unnecessary in most situations and only inflate the costs. The identification of microsatellite markers using NGS is a fast and cost-effective approach that is becoming economically feasible.

Thereby, DNA fingerprints generated with an adequately tailored set of markers, obtained by low-coverage sequencing of a well-selected representation of a genome, could remain the method of choice for most application areas in plant genotype identification especially in resource-limited developing countries. The term “DNA fingerprinting” via microsatellite hybridization once created by Alec Jeffreys could survive in the long run even in the era of brute force DNA sequencing, allowing the developing countries with limited financial and technological resources to effectively crush the biopiracy and corporate hijacking of their natural resources [54].

### 5.3. Establishment of International Barcode Certification

Establishing national framework for the natural product certification would be one of the reliable solutions for this malignant problem resulting from distributional conflicts between private users and provider countries. Proper identification method should be further developed into a standard certification assessed case by case. When highly processed, multi-ingredient herbal products that are considered DNA metabarcoding will be the useful analytical tool in authentication where microsatellite-based barcoding for raw natural products followed by metabarcoding for the end product should be implemented [61, 62].

Sri Lanka as a country with a heritage of its own traditional medical system possesses the knowledge of indigenous communities that can be integrated in health care systems based on Western medicine. This would pave the way in implementing the concept of a One-World Medicine which is an ethically and socially acceptable alternative to forms of biopiracy. Most of the natural product exporting nations are lacking with the technological advancement due their Gross domestic product (GDP). Developing standard operating procedures (SOP) with the low-cost certification for the local use and export of plant and natural products will be helpful in mitigating the adulteration [63, 64].

Availability of an authenticated National List of Essential Medicines that include traditional formulas and herbal preparations will be crucial for countries with rich heritage of traditional medicine for the seamless integration of the concept One-World Medicine. This system has already been proposed in Thailand. The large sums of private and public money devoted to research and development and the scientific and technological advancements in medicine have not yet translated into many clinical products specifically traditional formulas and herbal preparations [62].

One of the most successful ways to translate clinical applications bench to bedside is through collaborations. The P4 among indigenous medical practitioner, biotechnology, pharmaceutical industries, universities, and national research centers could enable all the parties to jointly make decisions on which research projects to develop and the steps that should be taken to establish One-World Medicine [65, 66].

## 6. Conclusion

Several initiations were established by academic institutions, industries, and international organizations to abolish adulteration and biopiracy including bioprospecting protocols. Unfortunately, the established systems are not properly followed by the developing countries due to their unaffordability of technical cost and policies. Considering cost-effective technologies and user-friendly agreements like Private-Public-People partnership will aid more support to abolish biopiracy.

Moreover, it is worth highlighting that most developing countries are mainly agricultural-based economy, and the GDP of the developing countries could be increased by enhanced bioprospecting via introduction of cost-effective DNA fingerprinting technologies and systemic establishment of 4P, utilizing their unique natural resources and traditional indigenous knowledge and thus not being a pray of corporate hijacking.

## Figures and Tables

**Table 1:** Global biopiracy incidents with details.

Incident	Country of origin	Pirated country	Reference
Karawila (*Momordica charantia*)	South Asian countries including Sri Lanka	USA	[10]
Turmeric (*Curcuma longa*)	India	USA	[11]
Kothala Himbutu (*Salacia reticulata*)	Sri Lanka	Japan, USA, Europe	[12, 13]
Magul Karanda (*Pogamia glabra*)	Sri Lanka	Japan	[13]
Masbadda (*Gymnema sylvestre*)	Sri Lanka	Japan	[13]
Heen Bavila (*Sida cordifolia*)	Sri Lanka	Japan	[13]
Weniwalgeta (*Coscinium fenestratum*)	Sri Lanka	Japanese, European, and USA pharmaceutical manufacturers	[14]
Neem (*Azadirachta indica*)	India Nepal	EPO to US Department of Agriculture and the US-American firm W.R.	[15]
Enola Bean (*Phaseolus vulgaris*)	Mexico	USA	[16]
Rubber seeds (*Hevea brasiliensis*)	Brazil	England	[17, 18]
Hoodia plant (*Hoodia gordonii*)	Southern Africa	CSIR gave patent to Phytopharm and Pfizer	[19]
Sacks of plant specimens	Philippine indigenous people	Philippine National Museum	[14]
Kakadu Plum (*Terminalia ferdinandiana*)	Australian Aboriginal people	USA	[3]
*Aloe vera*	Sri Lanka	USA	[13]

**Table 2:** Available genetic barcoding methods for the prevention of biopiracy.

Methods	Advantages	Disadvantages	References
Next-generation sequencing (NGS)	(i) No prior sequence information or probe generation is required	(i) High in cost and not available in all laboratories (ii) Time-consuming and high data store required	[55]
Simple sequence repeats (SSR) and intersimple sequence repeats (ISSR)	(i) Readily use for taxonomic studies	(i) Allele sizes often differ from different laboratories Lack of potential in discriminating species in hypervariables (ii) Occurrence of null alleles complicate the interpretation of data because heterozygotes cannot be identified, and reaction failures cannot be detected	[56, 57]
Amplified fragment length polymorphism (AFLP)	(i) No prior sequence information or probe generation is needed	(i) AFLP generates huge quantities of information technically demanding in the laboratory and, especially, in data analysis (ii) Interspecies genetic hybrids in the genus are difficult to identify	[58]
Random amplified polymorphic DNA (RAPD)	(i) Producing DNA patterns that allow comparison of many loci simultaneously (i) Low cost	(i) It may not be practical to identify the species of origin in products containing mixtures of species (ii) It does not seem to be adequate for analysis of severely degraded material, as in autoclaved samples	[59, 60]

## Data Availability

The data used to support the findings of this study are available from the corresponding author on request.

## Abbreviations

AFLP: Amplified fragment length polymorphism
CAPS: Cleaved amplified polymorphic sequence
CSIR: Council of scientific and industrial research
DAMD: Directed amplification of minisatellite DNA
EPO: European patent office
GDP: Gross domestic product
IRAP: Inter-retrotransposon amplified polymorphism
ISSR: Intersimple sequence repeats
ITS2: Internal transcribed spacer
NGS: Next-generation sequencing
RAPD: Random amplified polymorphic DNA
PPP: Private-Public partnership
REMAP: Retrotransposon-microsatellite amplified polymorphism
SCAR: Segments with chromatin alterations reinforcing senescence
SNPs: Single nucleotide polymorphisms
SRAP: Sequence-related amplified polymorphism
SSR: Simple sequence repeats
WIPO: World Intellectual Property Organization
4P: Private-Public-People partnership.
